# Orogenital Transmission of *Neisseria meningitidis* Causing Acute Urethritis in Men Who Have Sex with Men

**DOI:** 10.3201/eid2501.171102

**Published:** 2019-01

**Authors:** Arnaud Jannic, Hedi Mammeri, Lise Larcher, Vincent Descamps, William Tosini, Bao Phung, Yazdan Yazdanpanah, Fabrice Bouscarat

**Affiliations:** Hôpital Bichat Claude Bernard, Paris, France (A. Jannic, H. Mammeri, L. Larcher, V. Descamps, F. Bouscarat);; Université Paris Diderot, Paris (A. Jannic, H. Mammeri, V. Descamps, W. Tosini, B. Phung, Y. Yazdanpanah, F. Bouscarat);; Institut National de la Santé et de la Recherche Médicale, Paris (H. Mammeri)

**Keywords:** urethritis, Neisseria meningitidis, bacteria, orogenital transmission, whole-genome sequencing, sequence type 11, ST11, clonal complex, monogamous couple, men who have sex with men, sexually transmitted infections

## Abstract

*Neisseria meningitidis* sequence type 11 is an emerging cause of urethritis. We demonstrate by using whole-genome sequencing orogenital transmission of a *N. meningitidis* sequence type 11 isolate causing urethritis in a monogamous couple of men who have sex with men. These results suggest dissemination of this clonal complex among low-risk patients.

*Neisseria meningitidis* is a gram-negative diplococcus that can cause severe invasive infections. It usually colonizes the oropharynx and spreads through close or prolonged contact with respiratory or throat secretions. Cases of *N. meningitidis*–associated urethritis have been reported since the 1930s, and orogenital sex has been demonstrated as a potential mode of transmission by genotyping methods (*1*).

A 22-year-old man visited a sexual health clinic for symptomatic urethritis with purulent urethral discharge and pain while urinating. Clinical examination showed no other signs or symptoms, including fever. His last sexual intercourse (including orogenital contacts) occurred 3 weeks before with his stable, unique, asymptomatic male partner. Both men reported that they had not had any other sexual partner before and since their first intercourse together <1 year before. Meningococcal vaccination statuses were unknown.

Results of nucleic acid amplification testing for *N. gonorrhoeae* and *Chlamydia trachomatis* in urine, pharyngeal, and anal samples, and serologic testing for HIV, hepatitis B and C viruses, and syphilis were negative for the patient and his partner. Urethral culture yielded growth with oxidase and Gram stain results consistent with gonococcus, but matrix-assisted laser desorption/ionization time-of-flight mass spectrometry identified *N. meningitidis* (isolate TUR1). A pharyngeal swab specimen was collected from the partner of the patient to identify a potential source of contamination. This specimen also grew *N. meningitidis* (isolate CHA1). Isolates were nongroupable by slide agglutination serogrouping.

We purified genomic DNAs by using a NucleoSpin Tissue Kit (Macherey-Nagel, http://www.mn-net.com), prepared DNA libraries by using the Nextera Library Construction Kit (Illumina, https://www.illumina.com), and sequenced these DNAs by using a MiSeq sequencer (Illumina), which yielded 62× coverage for isolate TUR1 and 66× coverage for isolate CHA1. We assembled the genomes of CHA1 and TUR1 isolates by using SPAdes version 3.10.1 (http://bioinf.spbau.ru/spades), which resulted in assemblies consisting of 395 contigs for TUR1 and 636 contigs for CHA1 and yielded draft genomes of ≈2.19 Mb. We performed genome annotation by using the MicroScope Platform (http://www.genoscope.cns.fr/agc/microscope), which yielded 2,597 coding sequences, including protein-encoding genes and RNAs.

We analyzed high-throughput sequencing data by using the PubMLST website (https://pubmlst.org) for typing the CHA1 and TUR1 isolates. These 2 isolates belonged to the clonal complex sequence type (ST) 11 and had fine-type PorA 1.5,2; FetA F1-1. DNA sequence analysis of the *csw* gene of these isolates showed an open reading frame of 3,114 bp that had 99.9% nucleotide identity with that of the *N. meningitidis* W:2a:P1.7–2,4 ST11 strain (GenBank accession no. EU164779), which was consistent with genogroup W (*2**,**3*).

We used CSI Phylogeny version 1.4 (https://cge.cbs.dtu.dk/services/CSIPhylogeny) for phylogenomic analysis on the basis of single-nucleotide polymorphisms (SNPs) and called among core genomes of CHA1 and TUR1 isolates and 3 other ST11 *N. meningitidis* strains (NM3688 from Brazil [Bioproject PRJNA264543], COL-201505-31 from the United States [BioProject PRJNA324131]), and LNP27256 from France [Bioproject PRJNA215157]). Analysis showed that the CHA1 and TUR1 isolates differed by only 3 SNPs, thus demonstrating their clonal relationship. Conversely, these isolates differed by >890 SNPs from the other strains.

Nucleotide sequence accession numbers for whole-genome shotgun projects have been deposited at DNA Data Bank of Japan/European Molecular Biology Laboratory/GenBank under accession nos. MULP000000001 (Bioproject PRJNA369721) and MULO000000001 (Bioproject PRJNA369166).

The patient recovered quickly after receiving single doses of intramuscular ceftriaxone (500 mg) and azithromycin (1 g). His partner received a single dose of intramuscular ceftriaxone (500 mg) as a decontamination strategy.

Albeit rare, *N. meningitidis*–associated urethritis is emerging worldwide among men (*4**,**5*). These infections can be highly symptomatic and mimic gonococcal urethritis. Orogenital transmission is often suspected but rarely proven (*1*). In our study, use of whole-genome sequencing strongly supported the hypothesis that the oropharyngeal carriage of *N. meningitidis* by the man who performed oral sex was the source of the urethritis. Moreover, whole-genome sequencing showed that the strains belonged to the ST11 clonal complex. Several studies reported nongonococcal urethritis caused by *N. meningitidis* ST11 in Japan and the United States (*1**,**6**,**7*). These worldwide descriptions suggest that *N. meningitidis* S11 might represent an emerging urethrotropic clade.

This case is notable because the patient had highly symptomatic urethritis without risky sexual behavior. In fact, he reported that he and his only sexual partner had not had sex with others before they met.

Since 2010, several clusters of serogroup C invasive meningococcal disease have been reported among men who have sex with men, and sexual transmission is suspected to be involved (*8**–**10*). These infections led to an extension of the meningococcal vaccine recommendations to men who have sex with men who are engaged in risky behavior in some outbreaks areas. Our case highlights that sexual transmission of *N. meningitidis* should be considered for all men.

Finally, although systematically collected information is limited, meningococcal urogenital infections are potentially increasing and raising public health concerns. These infections need to be monitored, and bacteriological culture of purulent exudate should always be considered when available. Also, because fidelity might be contested between partners when a sexually transmitted disease is being diagnosed, identification of meningococcal urethritis and its transmission might have a strong psychological effect on the couple.
